# (*E*)-*N*′-(4-Hy­droxy­benzyl­idene)-2-nitro­benzohydrazide

**DOI:** 10.1107/S1600536810022075

**Published:** 2010-06-16

**Authors:** Shi-Yong Liu, Zhonglu You

**Affiliations:** a College of Chemistry & Pharmacy, Taizhou University, Taizhou Zhejiang 317000, People’s Republic of China; b Department of Chemistry, Liaoning Normal University, Dalian 116029, People’s Republic of China

## Abstract

In the title compound, C_14_H_11_N_3_O_4_, the two benzene rings form a dihedral angle of 45.3 (3)°. The nitro group is twisted out of the attached ring by a dihedral angle of 37.5 (3)°. In the crystal structure, mol­ecules are linked into a two-dimensional network parallel to (100) by O—H⋯O and N—H⋯O hydrogen bonds.

## Related literature

For the medicinal applications of hydrazone compounds, see: Hillmer *et al.* (2010 ▶); Zhu *et al.* (2009 ▶); Jimenez-Pulido *et al.* (2008 ▶); Raj *et al.* (2007 ▶); Zhong *et al.* (2007 ▶). For the crystal structures of hydrazones, see: Khaledi *et al.* (2009 ▶); Warad *et al.* (2009 ▶); Back *et al.* (2009 ▶); Vijayakumar *et al.* (2009 ▶). For related structures, see: Cao (2009 ▶); Xu *et al.* (2009 ▶); Shafiq *et al.* (2009 ▶).
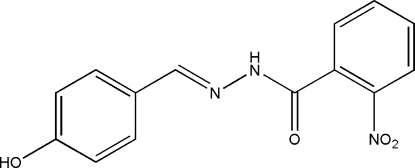


## Experimental

### 

#### Crystal data

C_14_H_11_N_3_O_4_
*M* *_r_* = 285.26
Orthorhombic, 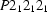

*a* = 7.720 (2) Å
*b* = 11.398 (3) Å
*c* = 15.072 (5) Å
*V* = 1326.3 (7) Å^3^
*Z* = 4
Mo *K*α radiation
μ = 0.11 mm^−1^
*T* = 298 K
0.20 × 0.18 × 0.18 mm

#### Data collection

Bruker SMART CCD area-detector diffractometer
Absorption correction: multi-scan (*SADABS*; Bruker, 2001 ▶) *T*_min_ = 0.979, *T*_max_ = 0.981
7745 measured reflections
1602 independent reflections
961 reflections with *I* > 2σ(*I*)
*R*_int_ = 0.085

#### Refinement

*R*[*F* ^2^ > 2σ(*F* ^2^)] = 0.048
*wR*(*F* ^2^) = 0.125
*S* = 1.02
1602 reflections
194 parameters
1 restraint
H atoms treated by a mixture of independent and constrained refinement
Δρ_max_ = 0.25 e Å^−3^
Δρ_min_ = −0.18 e Å^−3^

### 

Data collection: *SMART* (Bruker, 2007 ▶); cell refinement: *SAINT* (Bruker, 2007 ▶); data reduction: *SAINT*; program(s) used to solve structure: *SHELXTL* (Sheldrick, 2008 ▶); program(s) used to refine structure: *SHELXTL*; molecular graphics: *SHELXTL*; software used to prepare material for publication: *SHELXTL*.

## Supplementary Material

Crystal structure: contains datablocks global, I. DOI: 10.1107/S1600536810022075/ci5099sup1.cif

Structure factors: contains datablocks I. DOI: 10.1107/S1600536810022075/ci5099Isup2.hkl

Additional supplementary materials:  crystallographic information; 3D view; checkCIF report

## Figures and Tables

**Table 1:** Hydrogen-bond geometry (Å, °)

*D*—H⋯*A*	*D*—H	H⋯*A*	*D*⋯*A*	*D*—H⋯*A*
O1—H1⋯O2^i^	0.82	1.93	2.725 (3)	164
N2—H2*A*⋯O1^ii^	0.90 (1)	2.05 (2)	2.932 (4)	166 (4)

Symmetry codes: (i) 
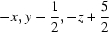
; (ii) 
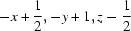
.

**Fig. 1.**
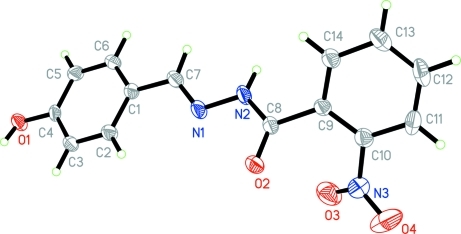
The molecular structure of the title compound. Displacement ellipsoids are drawn at the 30% probability level. Hydrogen atoms are shown as spheres of arbitrary radius.

**Fig. 2.**
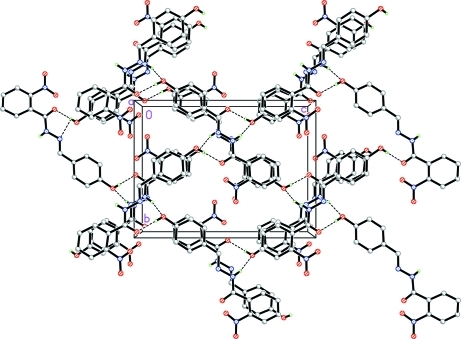
The molecular packing of the title compound, viewed along the a axis. Hydrogen bonds are shown as dashed lines. Hydrogen atoms not involved in hydrogen bonding have been omitted.

## supplementary crystallographic information

### Comment

Considerable attention has been focused on hydrazones and their medicinal applications (Hillmer *et al.*, 2010; Zhu *et al.*, 2009; Jimenez-Pulido *et al.*, 2008; Raj *et al.*, 2007; Zhong *et al.*, 2007). The study on the crystal structures of such compounds is of particular interest (Khaledi *et al.*, 2009; Warad *et al.*, 2009; Back *et al.*, 2009; Vijayakumar *et al.*, 2009). We report herein the crystal structure of the title new hydrazone.

In the title molecule (Fig. 1), the dihedral angle between the two benzene rings is 45.3 (3)°, indicating that the molecule is twisted. The dihedral angle between the N3/O3/O4 nitro group and the C9–C14 benzene ring is 37.5 (3)°. All the bond lengths are comparable to those observed in related structures (Cao, 2009; Xu *et al.*, 2009; Shafiq *et al.*, 2009).

In the crystal structure, molecules are linked through O—H···O and N—H···O hydrogen bonds, to form a two-dimensional network parallel to the (100) (Fig. 2 and Table 1).

### Experimental

The title compound was prepared by the condensation reaction of 4-hydroxybenzaldehyde (0.05 mol, 6 g) and 2-nitrobenzohydrazide (0.05 mol, 9 g) in anhydrous methanol (200 ml) at ambient temperature. Colourless block-shaped single crystals suitable for X-ray structure determination were obtained by slow evaporation of the methanol solution for a period of 8 d.

### Refinement

Atom H2A was located in a difference map and refined isotropically, with the N–H distance restrained to 0.90 (1) Å [U_iso_(H2) = 0.08 Å^2^]. The remaining H atoms were placed in idealized positions and constrained to ride on their parent atoms, with C–H distances of 0.93 Å, O–H distance of 0.82 Å, and with *U*_iso_(H) = 1.2*U*_eq_(C) and 1.5*U*_eq_(O). In the absence of significant anomalous dispersion effects, Friedel pairs were averaged.

### Crystal data

**Table:**

C_14_H_11_N_3_O_4_	*F*(000) = 592
*M**_r_* = 285.26	*D*_x_ = 1.429 Mg m^−^^3^
Orthorhombic, *P*2_1_2_1_2_1_	Mo *K*α radiation, λ = 0.71073 Å
Hall symbol: P 2ac 2ab	Cell parameters from 633 reflections
*a* = 7.720 (2) Å	θ = 2.5–24.5°
*b* = 11.398 (3) Å	µ = 0.11 mm^−^^1^
*c* = 15.072 (5) Å	*T* = 298 K
*V* = 1326.3 (7) Å^3^	Block, colourless
*Z* = 4	0.20 × 0.18 × 0.18 mm

### Data collection

**Table:**

Bruker SMART CCD area-detector diffractometer	1602 independent reflections
Radiation source: fine-focus sealed tube	961 reflections with *I* > 2σ(*I*)
graphite	*R*_int_ = 0.085
ω scans	θ_max_ = 26.6°, θ_min_ = 2.2°
Absorption correction: multi-scan (*SADABS*; Bruker, 2001)	*h* = −8→9
*T*_min_ = 0.979, *T*_max_ = 0.981	*k* = −14→13
7745 measured reflections	*l* = −18→18

### Refinement

**Table:**

Refinement on *F*^2^	Primary atom site location: structure-invariant direct methods
Least-squares matrix: full	Secondary atom site location: difference Fourier map
*R*[*F*^2^ > 2σ(*F*^2^)] = 0.048	Hydrogen site location: inferred from neighbouring sites
*wR*(*F*^2^) = 0.125	H atoms treated by a mixture of independent and constrained refinement
*S* = 1.02	*w* = 1/[σ^2^(*F*_o_^2^) + (0.0542*P*)^2^] where *P* = (*F*_o_^2^ + 2*F*_c_^2^)/3
1602 reflections	(Δ/σ)_max_ = 0.001
194 parameters	Δρ_max_ = 0.25 e Å^−^^3^
1 restraint	Δρ_min_ = −0.18 e Å^−^^3^

### Special details

**Table:**

Geometry. All esds (except the esd in the dihedral angle between two l.s. planes) are estimated using the full covariance matrix. The cell esds are taken into account individually in the estimation of esds in distances, angles and torsion angles; correlations between esds in cell parameters are only used when they are defined by crystal symmetry. An approximate (isotropic) treatment of cell esds is used for estimating esds involving l.s. planes.
Refinement. Refinement of F^2^ against ALL reflections. The weighted R-factor wR and goodness of fit S are based on F^2^, conventional R-factors R are based on F, with F set to zero for negative F^2^. The threshold expression of F^2^ > 2sigma(F^2^) is used only for calculating R-factors(gt) etc. and is not relevant to the choice of reflections for refinement. R-factors based on F^2^ are statistically about twice as large as those based on F, and R- factors based on ALL data will be even larger.

### Fractional atomic coordinates and isotropic or equivalent isotropic displacement parameters (Å^2^)

**Table:**

	*x*	*y*	*z*	*U*_iso_*/*U*_eq_	
N1	0.1071 (5)	0.7318 (3)	1.03691 (19)	0.0490 (10)	
N2	0.1251 (5)	0.7909 (3)	0.9568 (2)	0.0486 (9)	
N3	0.2475 (6)	1.1354 (4)	0.9364 (3)	0.0729 (13)	
O1	0.1478 (4)	0.3516 (2)	1.34410 (16)	0.0478 (8)	
H1	0.0826	0.3784	1.3820	0.072*	
O2	0.0109 (4)	0.9556 (2)	1.01482 (16)	0.0498 (8)	
O3	0.3197 (5)	1.0807 (4)	0.9939 (2)	0.0846 (12)	
O4	0.2255 (9)	1.2411 (4)	0.9389 (3)	0.151 (2)	
C1	0.1311 (5)	0.5519 (3)	1.1159 (2)	0.0386 (9)	
C2	0.0734 (5)	0.5986 (3)	1.1956 (2)	0.0415 (10)	
H2	0.0306	0.6749	1.1971	0.050*	
C3	0.0787 (5)	0.5334 (3)	1.2728 (3)	0.0411 (10)	
H3	0.0418	0.5657	1.3262	0.049*	
C4	0.1396 (5)	0.4197 (3)	1.2695 (2)	0.0362 (9)	
C5	0.1956 (5)	0.3714 (3)	1.1908 (3)	0.0428 (10)	
H5	0.2367	0.2948	1.1892	0.051*	
C6	0.1902 (5)	0.4375 (3)	1.1148 (2)	0.0428 (10)	
H6	0.2271	0.4047	1.0616	0.051*	
C7	0.1348 (6)	0.6220 (4)	1.0351 (2)	0.0452 (10)	
H7	0.1579	0.5856	0.9812	0.054*	
C8	0.0819 (5)	0.9035 (3)	0.9539 (2)	0.0406 (10)	
C9	0.1145 (5)	0.9615 (3)	0.8660 (2)	0.0385 (9)	
C10	0.1817 (6)	1.0730 (4)	0.8584 (3)	0.0470 (11)	
C11	0.1996 (6)	1.1296 (4)	0.7788 (3)	0.0570 (12)	
H11	0.2446	1.2052	0.7762	0.068*	
C12	0.1495 (7)	1.0721 (5)	0.7024 (3)	0.0649 (14)	
H12	0.1618	1.1088	0.6477	0.078*	
C13	0.0821 (6)	0.9617 (4)	0.7068 (3)	0.0609 (13)	
H13	0.0495	0.9228	0.6552	0.073*	
C14	0.0624 (5)	0.9078 (4)	0.7889 (2)	0.0468 (11)	
H14	0.0127	0.8336	0.7917	0.056*	
H2A	0.189 (5)	0.755 (4)	0.915 (2)	0.080*	

### Atomic displacement parameters (Å^2^)

**Table:**

	*U*^11^	*U*^22^	*U*^33^	*U*^12^	*U*^13^	*U*^23^
N1	0.076 (3)	0.0396 (19)	0.0313 (18)	0.0082 (19)	0.0090 (19)	0.0098 (15)
N2	0.073 (3)	0.040 (2)	0.0327 (19)	0.008 (2)	0.016 (2)	0.0108 (15)
N3	0.095 (4)	0.056 (3)	0.067 (3)	−0.013 (3)	0.011 (3)	−0.006 (2)
O1	0.065 (2)	0.0454 (16)	0.0334 (15)	0.0081 (15)	0.0097 (14)	0.0069 (12)
O2	0.068 (2)	0.0442 (16)	0.0377 (15)	0.0063 (16)	0.0144 (15)	−0.0023 (13)
O3	0.087 (3)	0.112 (3)	0.055 (2)	−0.020 (3)	−0.0063 (19)	−0.009 (2)
O4	0.262 (7)	0.056 (3)	0.133 (4)	−0.013 (3)	−0.004 (4)	−0.027 (3)
C1	0.049 (2)	0.033 (2)	0.034 (2)	0.001 (2)	0.0034 (19)	0.0029 (16)
C2	0.055 (3)	0.035 (2)	0.034 (2)	0.006 (2)	0.0031 (19)	0.0018 (17)
C3	0.051 (3)	0.041 (2)	0.032 (2)	0.002 (2)	0.0087 (18)	−0.0002 (18)
C4	0.041 (2)	0.038 (2)	0.0303 (19)	−0.007 (2)	0.0019 (18)	0.0099 (17)
C5	0.057 (3)	0.031 (2)	0.041 (2)	0.002 (2)	−0.001 (2)	−0.0005 (18)
C6	0.056 (3)	0.041 (2)	0.032 (2)	0.000 (2)	0.0040 (18)	−0.0034 (18)
C7	0.059 (3)	0.046 (2)	0.031 (2)	0.003 (2)	0.005 (2)	0.0022 (17)
C8	0.050 (3)	0.039 (2)	0.033 (2)	0.001 (2)	0.0062 (19)	0.0045 (18)
C9	0.047 (2)	0.034 (2)	0.034 (2)	0.009 (2)	0.0043 (19)	−0.0001 (17)
C10	0.053 (3)	0.044 (2)	0.043 (2)	0.008 (2)	0.008 (2)	0.001 (2)
C11	0.060 (3)	0.045 (3)	0.067 (3)	−0.003 (2)	0.013 (2)	0.019 (2)
C12	0.073 (3)	0.072 (3)	0.049 (3)	0.017 (3)	0.011 (3)	0.030 (3)
C13	0.073 (3)	0.074 (3)	0.036 (3)	0.013 (3)	0.000 (2)	0.004 (2)
C14	0.056 (3)	0.045 (2)	0.039 (2)	0.003 (2)	0.0006 (19)	0.0037 (19)

### Geometric parameters (Å, °)

**Table:**

N1—C7	1.270 (4)	C4—C5	1.377 (5)
N1—N2	1.389 (4)	C5—C6	1.372 (5)
N2—C8	1.327 (5)	C5—H5	0.93
N2—H2A	0.90 (1)	C6—H6	0.93
N3—O3	1.204 (5)	C7—H7	0.93
N3—O4	1.217 (5)	C8—C9	1.501 (5)
N3—C10	1.466 (6)	C9—C14	1.374 (5)
O1—C4	1.368 (4)	C9—C10	1.377 (5)
O1—H1	0.82	C10—C11	1.369 (5)
O2—C8	1.224 (4)	C11—C12	1.379 (6)
C1—C6	1.381 (5)	C11—H11	0.93
C1—C2	1.388 (5)	C12—C13	1.363 (7)
C1—C7	1.457 (5)	C12—H12	0.93
C2—C3	1.381 (5)	C13—C14	1.390 (5)
C2—H2	0.93	C13—H13	0.93
C3—C4	1.380 (5)	C14—H14	0.93
C3—H3	0.93		
			
C7—N1—N2	116.2 (3)	C1—C6—H6	119.4
C8—N2—N1	118.2 (3)	N1—C7—C1	121.3 (3)
C8—N2—H2A	123 (3)	N1—C7—H7	119.3
N1—N2—H2A	117 (3)	C1—C7—H7	119.3
O3—N3—O4	123.7 (5)	O2—C8—N2	123.9 (3)
O3—N3—C10	119.1 (4)	O2—C8—C9	121.5 (3)
O4—N3—C10	117.2 (5)	N2—C8—C9	114.4 (3)
C4—O1—H1	109.5	C14—C9—C10	116.8 (4)
C6—C1—C2	118.6 (3)	C14—C9—C8	120.0 (4)
C6—C1—C7	120.1 (3)	C10—C9—C8	123.0 (3)
C2—C1—C7	121.3 (3)	C11—C10—C9	123.2 (4)
C3—C2—C1	120.9 (3)	C11—C10—N3	116.0 (4)
C3—C2—H2	119.6	C9—C10—N3	120.7 (4)
C1—C2—H2	119.6	C10—C11—C12	118.6 (4)
C4—C3—C2	119.0 (3)	C10—C11—H11	120.7
C4—C3—H3	120.5	C12—C11—H11	120.7
C2—C3—H3	120.5	C13—C12—C11	120.4 (4)
O1—C4—C5	117.8 (3)	C13—C12—H12	119.8
O1—C4—C3	121.3 (3)	C11—C12—H12	119.8
C5—C4—C3	120.9 (3)	C12—C13—C14	119.5 (4)
C6—C5—C4	119.4 (3)	C12—C13—H13	120.2
C6—C5—H5	120.3	C14—C13—H13	120.2
C4—C5—H5	120.3	C9—C14—C13	121.6 (4)
C5—C6—C1	121.2 (4)	C9—C14—H14	119.2
C5—C6—H6	119.4	C13—C14—H14	119.2

### Hydrogen-bond geometry (Å, °)

**Table:**

*D*—H···*A*	*D*—H	H···*A*	*D*···*A*	*D*—H···*A*
O1—H1···O2^i^	0.82	1.93	2.725 (3)	164
N2—H2A···O1^ii^	0.90 (1)	2.05 (2)	2.932 (4)	166 (4)

Symmetry codes: (i) −*x*, *y*−1/2, −*z*+5/2; (ii) −*x*+1/2, −*y*+1, *z*−1/2.
